# Structure of the SWI/SNF complex bound to the nucleosome and insights into the functional modularity

**DOI:** 10.1038/s41421-021-00262-5

**Published:** 2021-04-27

**Authors:** Zhenyu He, Kangjing Chen, Youpi Ye, Zhucheng Chen

**Affiliations:** 1 Key Laboratory for Protein Sciences of Ministry of Education, School of Life Science, Tsinghua University, Beijing 100084, China; 2 Beijing Advanced Innovation Center for Structural Biology and Beijing Frontier Research Center for Biological Structure, School of Life Science, Tsinghua University, Beijing 100084, China; 3 Tsinghua-Peking Center for Life Sciences, Beijing 100084, China

**Keywords:** Cryoelectron microscopy, Chromatin remodelling

Dear Editor,

SWI/SNF is the prototype chromatin remodeling complex and regulates the expression of 5% genes in yeast^1^. It shows strong homology to RSC in yeast and the BAF complex in human cells. Within SWI/SNF, Snf2 functions as the ATP-dependent motor that drives the fundamental DNA translocation reaction underlying chromatin remodeling, whereas many of the auxiliary subunits target the enzyme to specific loci of the genome. When this manuscript is under preparation, the structure of the SWI/SNF complex bound to the nucleosome was reported^2^, in which many elements involved in nucleosome binding were poorly defined because of the modest resolution. To understand the mechanism of nucleosome recognition, we report the cryo-EM structure of the yeast SWI/SNF complex in complex with the mononucleosome.

We reconstituted the 12-subunit SWI/SNF complex in vitro by overexpressing the individual subunits in *E. coli* using the modified EcoExpress system^3^ (Supplementary Fig. S1). To facilitate protein expression, truncated forms of Swi1 (residues 251–1336) and Snf2 (residues 430–1400) were used. The structure of the SWI/SNF–NCP complex was determined at an overall resolution of 6.9 Å, with a local resolution of 3.1 Å at the nucleosome bound with finger helix (FH) of Snf5, and a local resolution of 3.6 Å at the substrate recruitment module (SRM) (Fig. 1a; Supplementary Fig. S2). The recombinant SWI/SNF complex bound to the nucleosome in a manner similar to that of the endogenous complex^2^ (Supplementary Fig. S3), supporting the complex was reconstituted into the active form. The current higher-resolution map allowed us to identify several functionally important elements, such as the FH of Snf5 and a YEATS-like domain of Snf12 (Supplementary Fig. S4a–f; more discussion below).

**Fig. 1:**
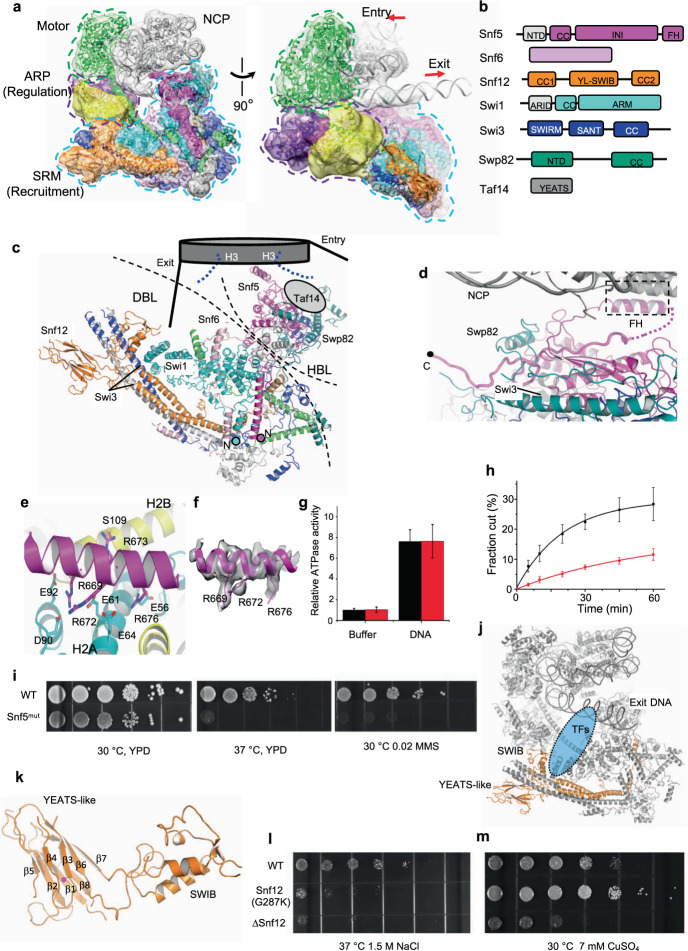
Structure of the SWI/SNF–NCP complex. **a** Two different views of the cryo-EM density superimposed with the structure of the SWI/SNF–NCP complex, showing the overall modularity (motor-regulation-recruitment) of the complex. Arrows indicate the directions of DNA translocation suggested by the binding position of the motor (Snf2). **b** Domain organization of the subunits within the SRM. YL-SWIB, the topologically linked YEATS-like and SWIB domain. **c** Structure of the SRM of the SWI/SNF complex. The proposed position of Taf14 subunit is indicated by the gray oval. The N-termini of Snf5 and Swi1 are labeled. **d** Structure of the C-terminal tail of Snf5. The boxed region is further analyzed in **e**. **e** Binding of the FH of Snf5 to the acidic pocket of H2A–H2B. **f** Local EM density map of the FH. **g** Relative ATPase activities of WT (black) and Snf5 mutant (red). Error bars indicate SD of the mean (*n* = 3 independent experiments). **h** Nucleosome remodeling activities of WT (black) and Snf5 mutant (red). Error bars indicate SD of the mean (*n* = 3 independent experiments). **i** Growth assays of yeast cells with WT and Snf5 mutant. All assays were performed in triplicate, and the representative ones are shown. **j** Structure of Snf12 (orange) within the NCP-bound SWI/SNF complex (gray). **k** Structures of the YEATS-like and SWIB domains. Arg126 is marked as a magenta dot. **l** The Snf12 mutation (G287K) resulted in sodium chloride sensitivity. **m** The Snf12 mutation (G287K) conferred copper sulfate resistance.

Based on the specific biological activities involved, the SWI/SNF complex is delineated into three functional modules: the motor module, the actin-related protein (ARP) module, and SRM (Fig. 1a). The Snf2 motor serves as the major gripping point on the nucleosome substrate and provides the driving force for DNA translocation^4^, whereas the regulatory ARP module binds to and stabilizes the helical conformation of the HSA helix, which directly contacts the motor and regulates the remodeling activity^5^. Most of the auxiliary subunits are assembled into the SRM, which is further delineated into two lobes (Fig. 1b, c), the DNA-binding lobe (DBL), and the histone-binding lobe (HBL). These auxiliary subunits recognize different features of the DNA and histone components of the nucleosome, and collectively provide the structural basis for chromatin targeting of the complex. Recently, the structures of several SWI/SNF family complexes are reported, and the architecture of which is defined in widely different ways^2,6–8^. We found that the tripartite organization principle of motor-regulation-recruitment also applies to RSC and BAF (Supplementary Fig. S5), which provides a unified theme to understand the functional modularity of these complexes.

DBL of SWI/SNF is composed of multiple elements, including Snf6, the N-termini of Swi1 and Snf5, and Snf12, which directly or indirectly bind to the DNA (Fig. 1c). Snf6 is crosslinked to the nucleosomal DNA^9^ and is in close proximity to the exit DNA. The N-termini of Swi1 and Snf5 interact with each other (Supplementary Fig. S4b), which may bind to the DNA of nucleosome either through the ARID domain directly, or indirectly through transcription coactivators^10^, providing the structural basis for their cooperation in SWI/SNF targeting. Snf12 interacts with the DNA-binding transcription factors through the SWIB domain^11^. Because the DBL is positioned close to the exit DNA (Fig. 1c), the structure suggests that different transcription factors interact with the DBL to recruit the SWI/SNF complex, and the motor will then slide the nucleosome away from the transcription factor-binding sites (on the exit side), potentially opening up the promoter regions, consistent with the role of the SWI/SNF complex in gene activation.

HBL contains two important elements for histone binding, Snf5 and Taf14. Although the structure of Taf14 could not be resolved in the current map, it is extensively cross-linked to Snf5 and Swp82, with the majority of the crosslinking sites mapped to the HBL^12^, suggesting that Taf14 resides at the HBL (Fig. 1c), consistent with its role in binding to the H3 tails^13^.

The most featured element in HBL is Snf5. Different from the homologs Sfh1 and INI1, Snf5 has a long C-terminal tail trailing the FH. A fragment of the C-terminal tail was found, which folded back and sandwiched between Swi3 and Swp82 at the HBL (Fig. 1d). In the absence of the nucleosome, the FH of Snf5 forms a short helical structure and packs against the INI domain intramolecularly^14^. It could not, however, be clearly defined in the nucleosome-bound structure reported previously^2^. The current high-resolution map of the nucleosome bound by SWI/SNF enabled us to detect the FH of Snf5 (Fig. 1e, f). Nucleosome binding triggers conformational changes of the FH (Supplementary Fig. S4g), with the N-terminus adopting an extended structure and stretching out from the HBL, and the C-terminus forming additional helical turns and interacting with the acidic pocket of H2A–H2B. This interaction mode between Snf5 and the nucleosome is conserved with those found for Sfh1 and INI1^7,8^ (Supplementary Fig. S4h).

To obtain experimental evidence for the importance of nucleosome binding by the FH, we truncated the C-terminus of Snf5 (Snf5^mut^), and assayed its biochemical activities in vitro. Consistent with the structure, the mutation did not perturb the assembly of the complex (Supplementary Fig. S6a), and the mutant complex showed an ATPase activity comparable to that of the complex with wild-type Snf5 (WT) (Fig. 1g). Yet, relative to the WT complex, the mutant displayed a 5-fold lower remodeling activity under the physiological salt concentration (150 mM KCl) (Fig. 1h; Supplementary Fig. S6b). Interestingly, at a lower salt concentration (50 mM KCl), the mutant showed activities similar to the WT complex (Supplementary Fig. S6c, d), which is possibly due to the increased affinity of the motor to the nucleosome at the low salt condition, bypassing the requirement of FH for stable substrate binding. Likewise, yeast cells carrying Snf5^mut^ grew similarly as the WT strain under the YPD condition (Fig. 1i), but were sensitive to high temperature and the DNA damage reagent MMS, probably due to the defects in transcription activation of stress response genes.

Intriguingly, at the periphery of the DBL, we identified an IgG-fold domain of Snf12 (Fig. 1j). Because the SWIB domain inserts into the β6–β7 loop (Fig. 1k), the IgG-fold is barely detectable at the sequence level. Structural comparison indicated that this IgG-fold domain shows a strong analogy to the YEATS domain of TAF14 (Supplementary Fig. S7). This YEATS-like domain is ~9 nm away from the exit DNA without any steric hindrance, which would allow binding to transcription factors in a manner similar to the SWIB domain (Fig. 1j). To validate the function of the YEATS-like domain, we mutated the highly conserved residue Gly287 to lysine (G287K). The G287K mutation conferred sensitivity to the elevated salt condition at 37 °C, to a level similar to that caused by Snf12 deletion (Fig. 1l), consistent with the role of Snf12 in mediating gene regulation in stress response^11^. Interestingly, whereas yeast cells without Snf12 were sensitive to copper and cobalt as reported before^15^, the G287K mutation resulted in metal resistance (Fig. 1m; Supplementary Fig. S8). Multiple membrane transporters and protein traffic networks affect metal tolerance^15^, and the specific factor(s) that interact with the YEATS-like domain of Snf12 and mediate the metal tolerance response is currently unknown. Snf12 would probably not bind to the histone tails, as it lacks the characteristic aromatic cage motif for recognition of acetylated/crotonylated lysine (Supplementary Fig. S7b). It will be of interest to identify the downstream effectors in the future. This YEATS-like domain is strictly conserved among the Snf12/Rsc6/BAF60 family proteins (Supplementary Fig. S7c). Over 200 somatic mutations of BAF60 were reported in the cBioPortal database, and the mutation R183Q was found in multiple cases and considered to be a recurrent mutational hotspot. Arg183 is conserved and maps to β1 (Arg126) of the YEATS-like domain of Snf12 (Fig. 1k), mutation of which might perturb the structural integrity of the well-folded YEATS-like domain.

## Supplementary information

SI

## Data Availability

Density maps are deposited at the Electron Microscopy Database (accession codes: EMD-31137, EMD-31136, EMD-31106) and protein coordinates are deposited at the Protein Data Bank (PDB 7EGP, 7EGM, and 7EG6) for the SWI/SNF-NCP, SRM, and NCP-FH, respectively.
