# Supplementary material for: Structure of the SWI/SNF complex bound to the nucleosome and insights into the functional modularity
Source: Cell Discov. 2021 Apr 27;7:28. doi: 10.1038/s41421-021-00262-5 (PMC8079448; doi:10.1038/s41421-021-00262-5)
Supplement: Supplementary file 1 — SI [file 41421_2021_262_MOESM1_ESM.pdf]

Materials and Methods

Expression and purification of proteins

The full-length Swi3, Snf6, Snf12, Snf5, Taf14, Swp82, Arp7, Arp9, Snf11 and Rtt102 and two truncated ORFs (residues 251-1336 of Swi1, and 430-1400 of Snf2) were divided into 4 groups and subcloned into respective vectors. Group 1 contained six subunits: Swi3, Snf6, and Snf12, which were assembled into the pHV vector (Supplementary Fig. S1), and Snf5, Taf14, and Swp82, which were assembled into the pET15b-based vector using the EcoExpress system[1]. Group 2 contained three subunits: Snf2 in the first open-reading frame of the pCDFduet-1 vector, and Arp7 and Arp9 in the pET15b-based vector[1]. Group 3 contained 2 subunits: Swi1 and Snf11 in the pRSFduet vector. Group 4 contained Rtt102 in the pET28a vector. C-terminal His-tags were added to Swi3, Snf2 and Swp82, whereas C-terminal Strep- and Flag- tags were added to Swi1 and Snf5, respectively. Each group was separately expressed in *E. coli* (Rosetta) and induced by 0.5mM IPTG when the OD600 reached 0.8. After 12 hours, the four groups of cells were harvested and lysed together utilizing W buffer (20 mM Hepes, 500 mM KCl, 10% glycerol, 0.1% Tween-20, 2mM MgCl2, pH 7.5 with protease inhibitor cocktail) by high-pressure homogenizer (ATS). After centrifugation, DNA was first precipitated by PEI and the supernatant was then precipitated by ammonium sulfate. The final precipitate was dissolved by W buffer and loaded onto a Ni Hi-trap column (GE Health Care). The eluted sample was then subjected to Strep pull-down (IBA) and the purified SWI/SNF was released by adding 8 mM d-biotin (sigma) to B buffer (20 mM Hepes, 200 mM KCl, 10% glycerol, 0.1% Tween-20, 2mM $MgCl_2$, pH 7.5 with protease inhibitor cocktail). The complex was further purified by resource S (GE Health Care) and glycerol gradient. The peak fractions were concentrated to 1 mg/ml. The mutated SWI/SNF complex (containing residues 1-656 of SNF5) was purified in a similar way with an additional step of Flag-tag affinity chromatography.

Nucleosome preparations

Nucleosome for structure analysis containing a central "601" sequence with flanking sequences of 29-bp and 59-bp (29N59) was prepared as described before[2]. The DNA sequence is amplified using PCR and listed as 5'-strand:

5'-
TTATGTGATGGACCCTATACGCGGCCGCCCTGGAGAATCCCGGTGCCGAGGCCG
CTCAATTGGTCGTAGACAGCTCTAGCACCGCTTAAACGCACGTACGCGCTGTCCC
CCGCGTTTTAACCGCCAAGGGGATTACTCCCTAGTCTCCAGGCACGTGTCAGATA
TATACATCCTGAAGCTTGTCGAGAAGTACTAGAGGATCATAATCAGCCATACCAC
ATTTGTAGAGGTTTTA-3' (the 601 sequence is underscored).

ATPase assay and remodeling assay

ATPase and nucleosome remodeling activity were measured in a similar way described previously[3]. An EnzCheck Phosphate Assay Kit (Life Science) was used for ATPase assay. Protein (50 nM) was incubated with 167-bp DNA (200 nM) in 50 mM Tris-HCl, pH 7.5, 1 mM $MgCl_2$ and 150 mM NaCl at 30 ℃.

In the nucleosome remodeling assays, 5 nM protein was incubated with 5 nM cy5-labeled 347-bp nucleosome in under the similar conditions as described before[3]. To measure the remodeling activity at the physiological salt conditions, 20 nM protein was used in a remodeling buffer (20 mM Tris-HCl, pH 8.0, 150 mM KCl, 5 mM $MgCl_2$ and 0.1 mg ml^{-1} bovine serum albumin).

Snf5 and Snf12 genetics

To generate the $Snf5^{mut}$ strain, the C-terminal tail of Snf5 (residues 657-905) was replaced by a DNA fragment encoding 10*His and Protein A tag. The fragment also carried a Kamix gene, which enabled the strain to acquire the ability to grow on the G418 medium (YPD +G418). To generate the Snf12 (G287K) strain, WT Snf12 was first replaced by URA3, and the yeast cells were selected using SC/-Ura sodium medium. The URA3 gene was then replaced by Snf12 (G287K), and the cells were selected using 5-FOA sodium medium (YPD +5FOA). The yeast stain BY4741 (MATa *leu2 ura3 his3 met15 can1*) was used, and transformed with lithium acetate method. All the mutants were confirmed by PCR, and DNA sequencing.

To determine the effect of mutations on yeast growth under stress conditions, we conducted spot assay. All strains were cultured in YPD for 48 hours to the platform stage, diluted in YPD to the optical density OD600 of 0.1, and grew to 1 at 30 ℃. Ten-fold serial dilutions of the transformants were spotted on YPD plates for temperature sensitivity testing (plates grown at 30 ℃ and 37 ℃), and on drug plates (YPD + 0.022% MMS or YPD + 1.4 M NaCl) to check for sensitivities. The effects of metal toxicity were examined similarly. All assays were performed in triplicates.

CryoEM sample preparation

SWI/SNF was mixed with 29N59 NCP in a ratio of 1:2 in the presence of ADP-BeFx in a similar way described before[4]. After dialysis for 3 hours, the complex was subjected to Grafix treatment (0-0.25% glutaraldehyde). The peak fraction was concentrated for electron microscopy analysis.

Negative stain of the SWI/SNF-nucleosome complex was performed with 2% uranyl acetate. Grids were examined using an FEI T12 microscope operated at 120 kV, and

images were recorded using a 4k x 4k charge-coupled device (CCD) camera (UltraScan 4000, Gatan).

To prepare cryo-grids, Quantifoil gold R2/1 grids with 200 mesh size were subjected to glow discharge in air for 30 s using a PDC-32G-2 Plasma Cleaner set to a low power. Samples (4 μL at 1μM) were blotted for 3s at -2 force before being plunge-frozen in liquid ethane with a FEI Vitrobot IV at 8 ℃ and 100% humidity.

Grids were examined and screened using an FEI Tecnai Arctica operated at 200 kV.
Cryo-EM data were collected using an FEI Titan Krios operated at 300 kV equipped with a K3 direct electron detector and GIF Quantum energy filter (Gatan), at a nominal magnification of 81,000× for a final pixel size of 0.5371 Å/pixel with the defocus values ranging from -1.3 to -1.8 μm. The total electron dose was 50 $e^-/Å^2$ fractionated in 32 frames (exposure time 2.56s). AutoEMation II (developed by Jianlin Lei) was used for automated data collection.

Image processing

The initial 3D model of the SWI/SNF- nucleosome complex was reconstructed using Relion3.0[5]. A total of 32,592 dose-fractionated image stacks were aligned using MotionCor2 with twofold binning[6], resulting in a pixel size of 1.0742 Å/pixel, and the CTF parameters were estimated using CTFFIND4[7]. Particle picking, two-dimensional (2D) classification and three-dimensional (3D) classification were carried out in Relion3.0. The initial picked particles were extracted with fourfold binning (4.2968 Å/pixel) to increase signal to noise ratio. After multiple rounds of 2D classification, a total of 2550k particles were selected and subjected to further processing. Particles were then re-extracted with twofold binning (2.1484

Å/pixel) and a set of 229k particles were selected after 3D classification and yielded a structure of SWI/SNF-NCP at an overall resolution of 6.9 Å. The particles with clear features from the last round 3D classification were re-extracted without binning (1.0742 Å/pixel) and subjected to focused 3D classification (masking SRM and NCP-FH, respectively). After multi-round focused classification and auto-refinement, the SRM module of SWI/SNF were reconstructed at a resolution of 3.6 Å with 210k particles and the nucleosome module including the finger helix reached 3.1 Å with 525k particles.

Model building

The initial model was built by fitting the maps in Chimera using the known structures, including Snf2-nucleosome complex (PDB code 5Z3V)[4], SWI/SNF body module (PDB code 6UXV)[8] as the templates. The atomic models for the rest of the molecules were built manually in Coot. In building the finger helix of Snf5, the R669 and R676 are clearly defined and the helix orientation were further confirmed through the whole 6.9 Å map. The structures were refined using Phenix with secondary structure constrains.

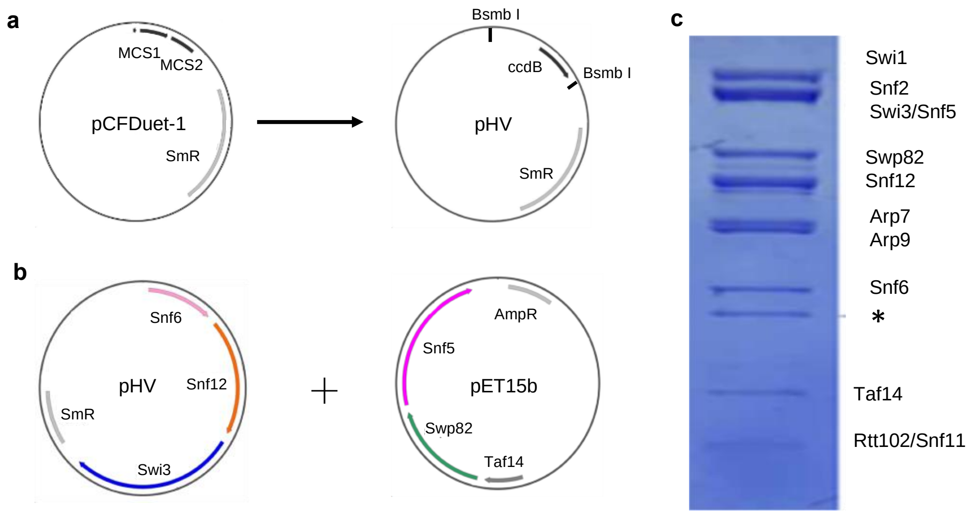

Supplementary Figure 1 | Reconstitution of the SWI/SNF complex in vitro (**a**) Engineering of the pCDFDuet-1 vector to make the pHV vector. (**b**) Strategies to co-express Swi3, Snf6, Snf12, Snf5, Taf14 and Swp82 using two compatible vectors. (**c**) SDS-PAGE gel of the SWI/SNF complex reconstituted in vitro. *, degradation product.

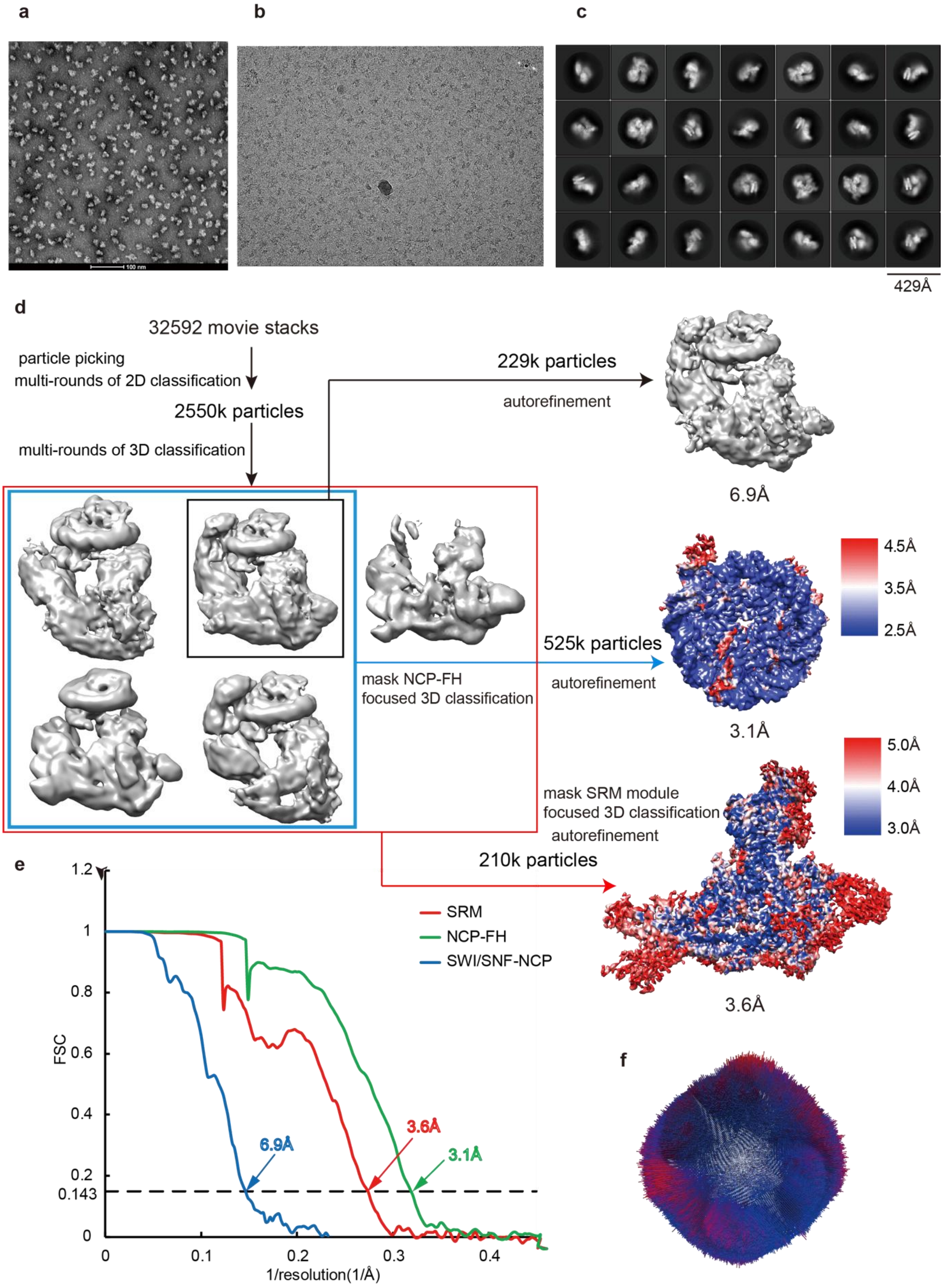

Supplementary Figure 2 | CryoEM analysis of the SWI/SNF-nucleosome complex.

(**a**) A representative negative stain micrograph

(**b**) A representative cryo-EM micrograph.

(**c**) 2D class averages of characteristic projection views of cryo-EM particles.

(**d**) Flowchart of the cryo-EM data processing for the SWI/SNF-nucleosome dataset.

(**e**) Resolution estimation of the EM maps. Gold standard Fourier shell correlation (FSC) curves, showing the nominal resolutions of 6.9 Å, 3.6 Å, and 3.1 Å for the whole complex, the substrate recruitment module, and the nucleosome region, respectively.

(**f**) Angular distributions of the cryo-EM particles in the final round of refinement of the SWI/SNF-NCP complex.

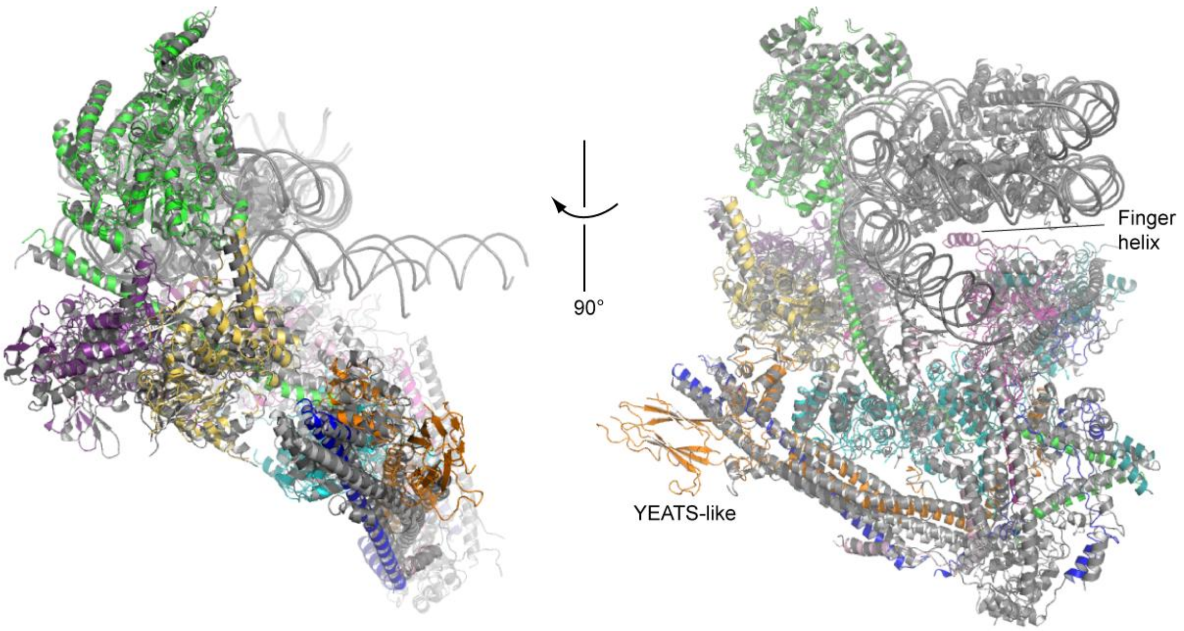

Supplementary Figure 3 | Structural comparison of the reconstituted and the endogenous SWI/SNF complexes. The reconstituted complex is colored as Fig. 1, and the endogenous complex (PDB code 6UXW) is in grey [8]. The structures of the bound nucleosome are aligned.

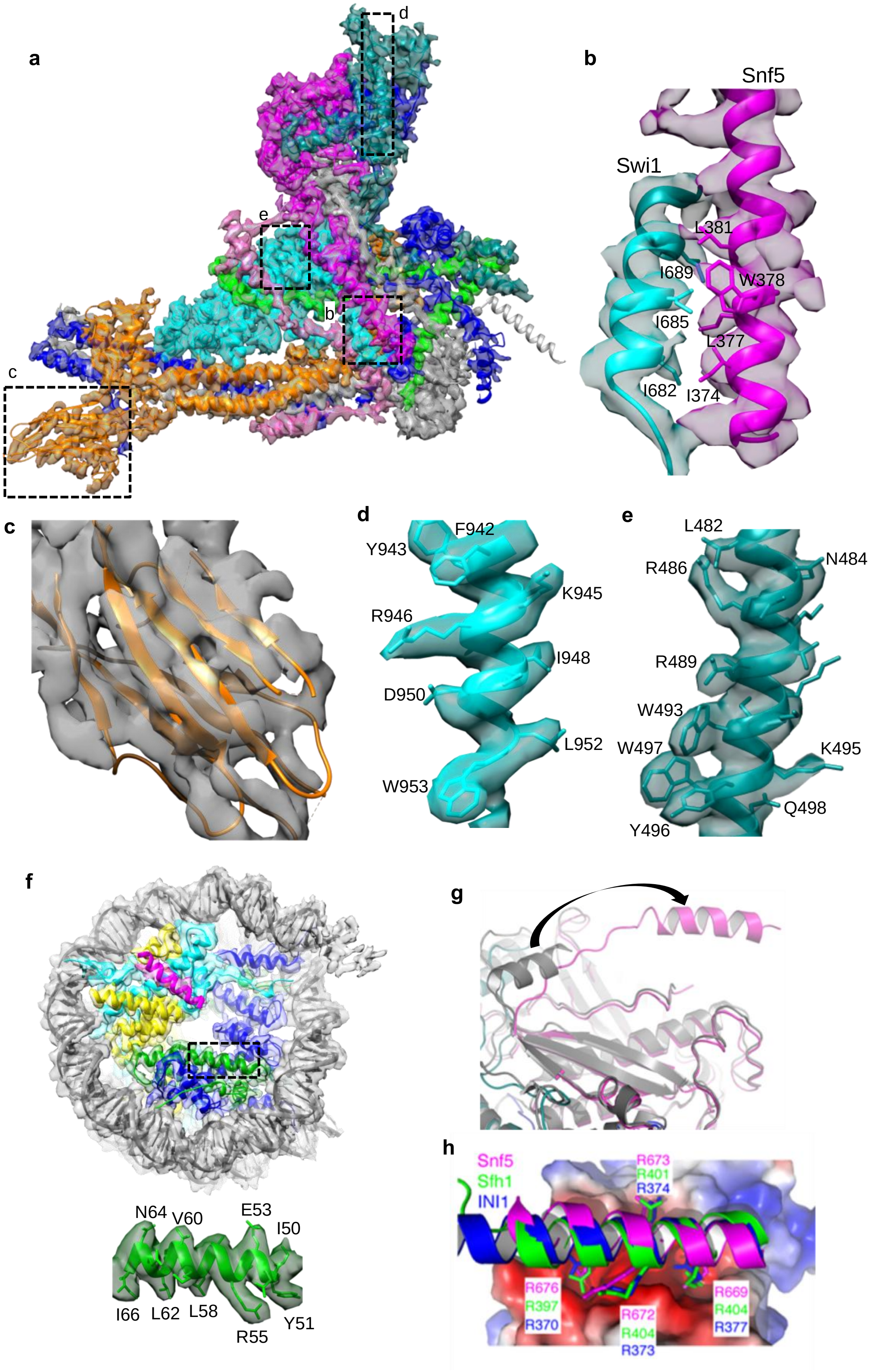
a
b
c
d
e
Snf5
Swi1
L381
I689
W378
I685
L377
I682
I374
F942
Y943
K945
R946
I948
D950
L952
W953
L482
R486
N484
R489
W493
W497
K495
Q498
Y496
f
g
h
Snf5
Sfh1
INI1
R673
R401
R374
R676
R397
R370
R672
R404
R373
R669
R404
R377
N64
V60
E53
I50
I66
L62
L58
R55
Y51

Supplementary Figure 4 | Local structural analysis of the SWI/SNF-nucleosome complex. (**a**) Overall EM density of SRM module. Snf2:green; Swi1:cyan; Snf5: magenta; Swi3:blue and gray; Snf12:orange; Snf6:plum; Swp82:dark cyan; Close-up views of the boxed region are shown. (**b**) Interaction between the N-terminal CCs of Snf5 (magenta) and Swi1 (cyan). (**c**) Local EM density of the YEATS-like domain of Snf12. (**d**) Local EM density of the helix (residues 942-953) of Swi1. (**e**) Local EM density of the CC of Swp82. (**f**) Overall EM density of the nucleosome bound with the FH of Snf5. The boxed region is enlarged and shown below. The close-up view of FH is shown in Figure. 1f. H3, blue; H4, green; H2A, cyan; H2B, yellow; FH, magenta. (**g**) Structural comparison of the FH with (color magenta) and without (grey, PDB code 7c4j)[9] binding to the nucleosome. The alignment is performed on the structure of the INI domain. The conformation change of FH is indicated by the arrow. (**h**) Comparison of the interactions of the finger helixes of Snf5 (magenta), Sfh1 (green, PDB code 6TDA) [3], and INI1 (blue, PDB code 6LTJ) [4] to the acidic pocket of H2A-H2B (surface electron penitential of H2A-H2B is calculated with Pymol).

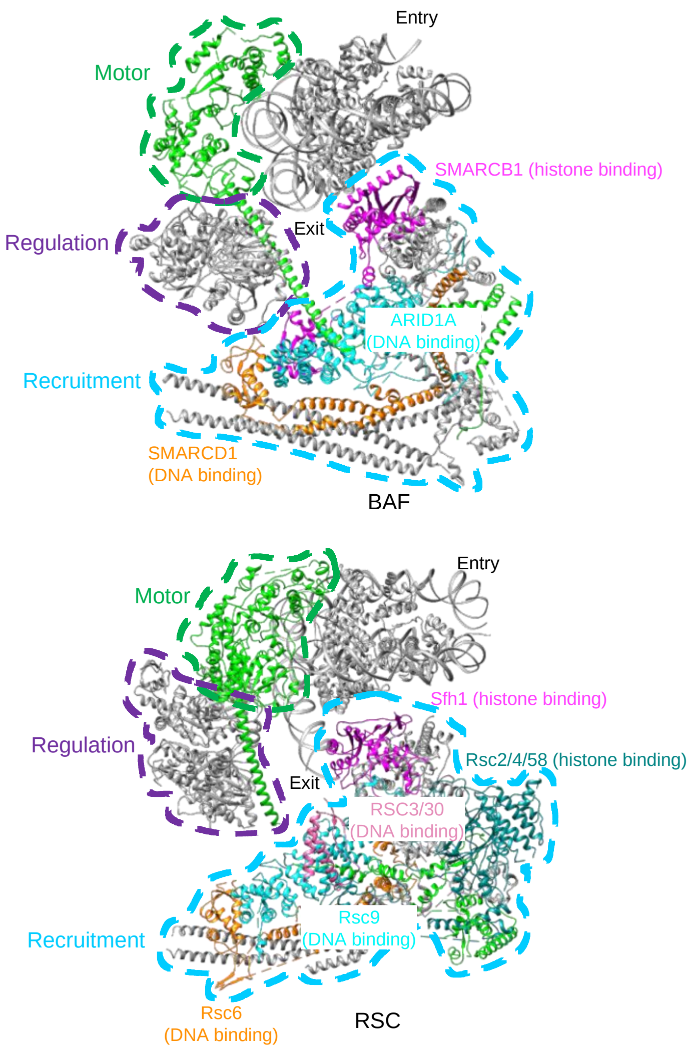

Supplementary Figure 5 | Motor-regulation-recruitment modularity of BAF and RSC. The models are based on PDB code 6LTJ for BAF[10], and PDB code 6KW3 for RSC[11]. The actin and actin-related proteins are organized into the regulation module: Arp7 and Arp9 in RSC and SWI/SNF, Actin and Arp4 in BAF. Subunits involved in histone binding: SMARCB1/Sfh1 (homology of Snf5 in SWI/SNF), Rsc2/4/58 (RSC specific). Subunits involved in DNA binding (directly or indirectly through transcription factors): ARID1A/Rsc9 (homology of Swi1 in SWI/SNF), SMARCD1/Rsc6 (homology of Snf12 in SWI/SNF), Rsc3/30 (RSC specific). These nucleosome-binding subunits are organized into the recruitment module.

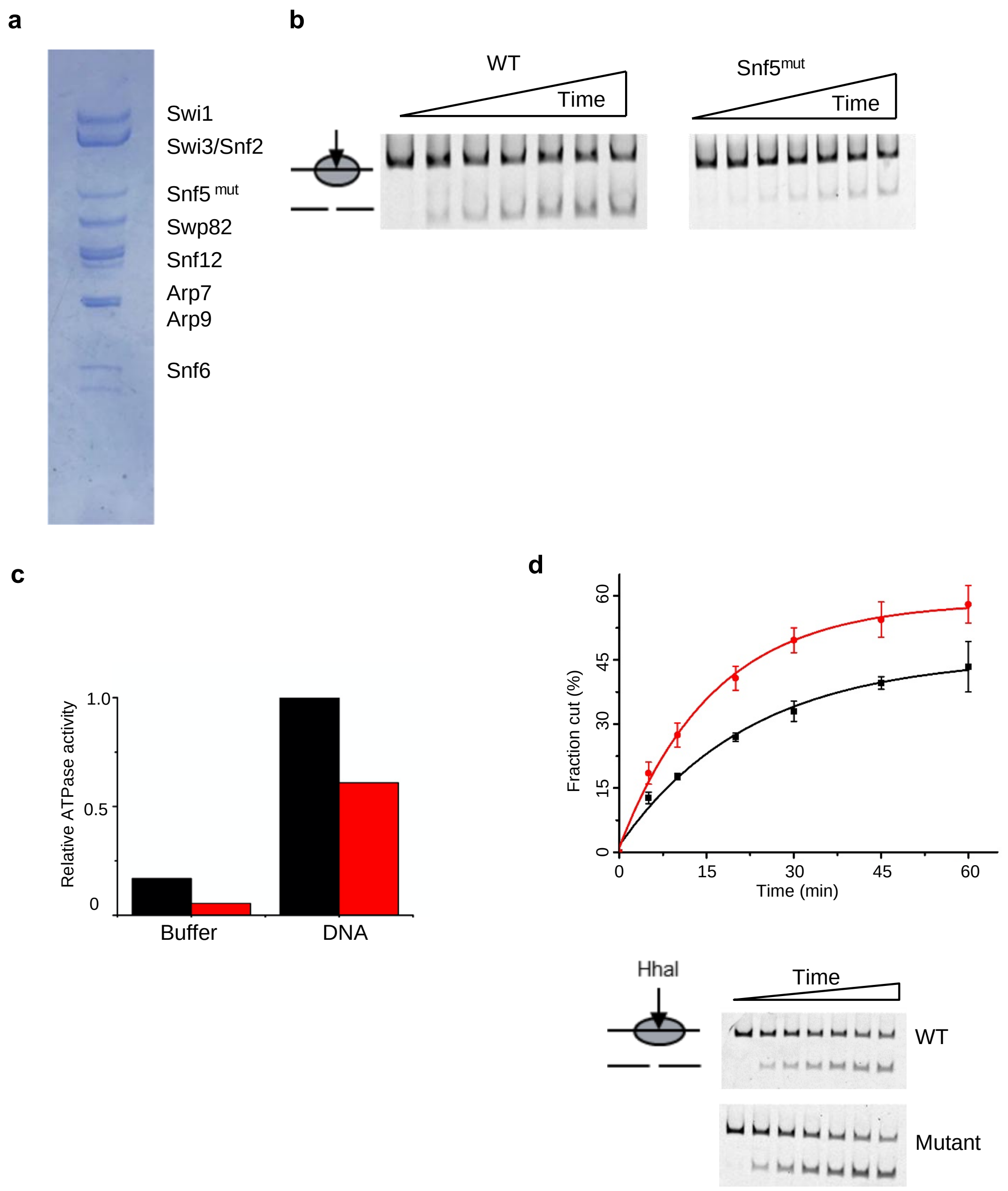

Supplementary Figure 6 | Biochemical analysis of the Snf5 mutant complex (**a**) SDS-PAGE gel of the SWI/SNF complex with the C-terminal truncation mutant Snf5 (residues 1-656). (**b**) Representative gels of the nucleosome remodeling activities of the WT and Snf5 mutant complexes at the physiological salt conditions (150 mM KCl). (**c**) Relative ATPase activities of the WT (black) and Snf5 mutant (red) complexes at the low salt conditions (50 mM KCl). (**d**) Nucleosome remodeling activities of the WT (black) and Snf5 mutant (red) complexes at the low salt conditions (50 mM KCl). Representative gels are shown at the bottom.

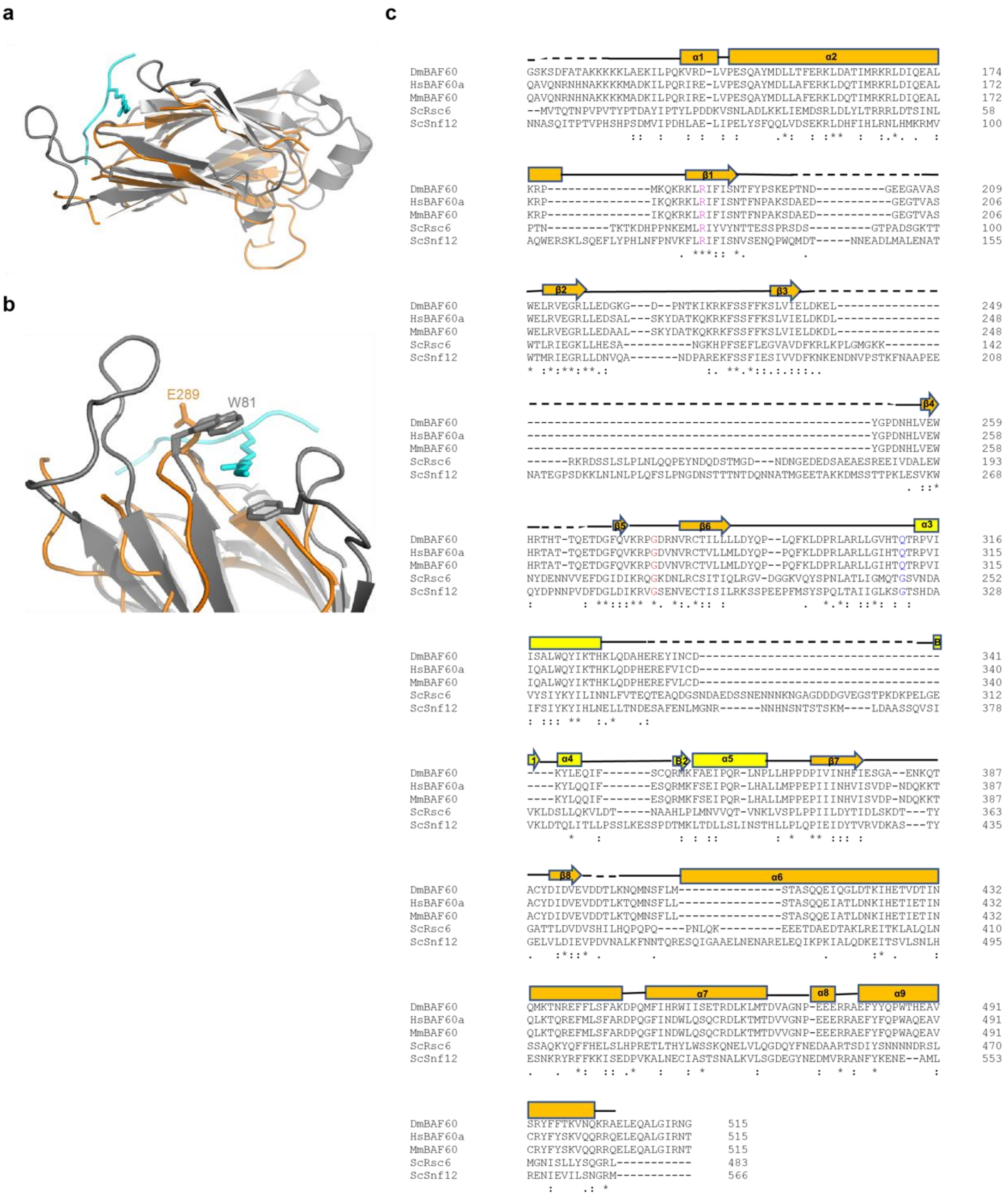

Supplementary Figure 7 | Sequence and structural analyses of Snf12 (**a**) Structural alignment of the YEATS-like domain of Snf12 with the YEATS domain of Taf14 (grey, PDB code 5iok)[12]. The H3 peptide bound by Taf14 is colored cyan. (**b**) Structural comparison of the YEATS-like domain of Snf12 and Taf14 (grey, the bound H3 in cyan, PDB code 5iok) [12] at the aromatic cage region. (**c**) Multi-sequence alignments of the Snf12 homologs. The inserted SWIB domain is colored yellow. The alignments are performed using the Clustal Omega sever.

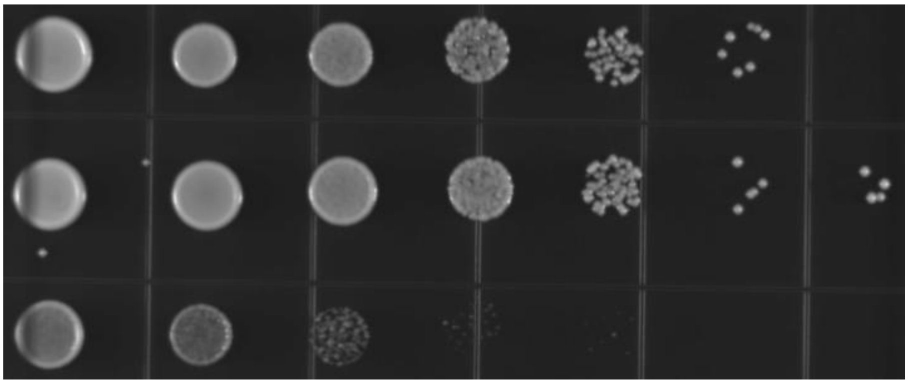

Supplementary Figure 8 | The Snf12 mutation (G287K) conferred cobalt sulfate resistance.

Additional references:

1 Qin, Y. *et al.* EcoExpress-Highly Efficient Construction and Expression of Multicomponent Protein Complexes in Escherichia coli. ACS Synth. Biol. 5, 1239-1246 (2016).
2 Xia, X., Liu, X., Li, T., Fang, X. & Chen, Z. Structure of chromatin remodeler Swi2/Snf2 in the resting state. Nat. Struct. Mol. Biol. (2016).
3 Liu, X., Li, M., Xia, X., Li, X. & Chen, Z. Mechanism of chromatin remodelling revealed by the Snf2-nucleosome structure. Nature 544, 440-445 (2017).
4 Li, M. *et al.* Mechanism of DNA translocation underlying chromatin remodelling by Snf2. Nature 567, 409-413 (2019).
5 Scheres, S. H. RELION: implementation of a Bayesian approach to cryo-EM structure determination. J. Struct. Biol. 180, 519-530 (2012).
6 Zheng, S. Q. *et al.* MotionCor2: anisotropic correction of beam-induced motion for improved cryo-electron microscopy. Nat. Methods 14, 331-332 (2017).
7 Rohou, A. & Grigorieff, N. CTFFIND4: Fast and accurate defocus estimation from electron micrographs. J. Struct. Biol. 192, 216-221 (2015).
8 Han, Y., Reyes, A. A., Malik, S. & He, Y. Cryo-EM structure of SWI/SNF complex bound to a nucleosome. Nature 579, 452-455 (2020).
9 Wang, C. *et al.* Structure of the yeast Swi/Snf complex in a nucleosome free state. Nat Commun 11, 3398 (2020).
10 He, S. *et al.* Structure of nucleosome-bound human BAF complex. Science 367, 875-881 (2020).
11 Ye, Y. *et al.* Structure of the RSC complex bound to the nucleosome. Science 366, 838-843 (2019).
12 Andrews, F. H. *et al.* The Taf14 YEATS domain is a reader of histone crotonylation. Nat. Chem. Biol. 12, 396-398 (2016).
